# T Cell Polarization at the Virological Synapse

**DOI:** 10.3390/v2061261

**Published:** 2010-05-31

**Authors:** Clare Jolly

**Affiliations:** MRC Centre for Medical Molecular Virology, Division of Infection and Immunity, University College London, W1T 4JF, UK; E-Mail: c.jolly@ucl.ac.uk; Tel.: +44-207-679-9558; Fax: +44-207-679-9550

**Keywords:** HIV, T cell, virological synapse, polarization, cytoskeleton, MTOC, secretion

## Abstract

Cell-to-cell spread of HIV-1 between CD4^+^ T cells takes place at multimolecular structures called virological synapses. A defining feature of the virological synapse is polarization of viral assembly and budding at sites of T cell-T cell contact. Recent work is beginning to address how viral proteins are targeted to the virological synapse and the molecular mechanisms that regulate HIV-1 egress by cell-to-cell spread. This review discusses our current understanding of these processes and considers how T cell polarization during other forms of intercellular communication may provide insight into HIV-1 assembly and dissemination.

## 1. Introduction

Direct cell-to-cell spread is a mechanism of viral transmission that is utilized by many viruses in order to promote rapid and efficient dissemination between target cells [1] and the Human Immunodeficiency Virus type-1 (HIV-1) is no exception. The idea that HIV-1 can disseminate by direct cell-to-cell spread, in addition to cell-free infection has been known for many years [2]. Early *in vitro* studies on HIV-1 infected peripheral blood mononuclear cells observed polarization of HIV-1 budding towards engaged target cells [3] and the localization of viral proteins, adhesion molecules and cytoskeletal elements at the leading edge of infected cells and at sites of cell-cell contact [2–7]. *In vivo,* foci of infected T cells and macrophages have been observed in lymphoid tissue [8,9] indicative of cell-cell spread. As the major site of early HIV-1 replication, the gut-associated lymphoid tissue (GALT) supports robust HIV-1 infection and therefore could represent an environment in which the close association of susceptible cells would favor cell-to-cell transmission [10–14] although the relative contribution of cell-cell and cell-free infection *in vivo* in the GALT remains to be demonstrated. Collectively, this work has led to the hypothesis that HIV-1 may exploit intercellular contacts to promote dissemination between permissive cells; however, it was not until relatively recently that the molecular mechanisms that regulate cell-to-cell spread were interrogated, leading to the first publication of a retrovirus-induced virological synapse (VS), that formed by HTLV [15]. HIV-1 induced VS have also been described between infected and uninfected CD4^+^ T cells [16,17], between dendritic cells and CD4^+^ T cells [18], between macrophages and CD4^+^ T cells [19,20] and between monocytes and epithelial cells [21]. Thus, direct cell-cell spread at VS appears to be a generalized feature of retroviral dissemination between immune cells.

*In vivo*, HIV-1 predominantly replicates in CD4^+^ T lymphocytes. Productive infection of T cells is mediated by binding of the HIV-1 envelope glycoprotein (Env) subunit gp120 to CD4 and coreceptor (either CXCR4 or CCR5) on the surface of target cells (reviewed in [22]). Receptor binding triggers a conformational change in Env that exposes the gp41 fusion peptide and brings the viral and cellular membranes into close apposition, culminating in fusion, translocation of the viral core containing the HIV-1 genome into the cytoplasm and eventual integration of the viral DNA into the host genome. HIV-1 replicates in activated CD4^+^ T cells and the production of nascent virions leads to dissemination either by cell-free means or by direct cell-to-cell spread. Current work is beginning to elucidate the spatio-temporal recruitment of viral and cellular proteins to sites of cell-cell contact and to probe cellular mechanisms that orchestrate polarized HIV-1 egress from T cells. In this review, I will discuss on how the process of polarization within the HIV-1 infected T cell during T cell-T cell contact may contribute to orchestrating viral assembly and transmission at the VS. Readers are directed to the review by Vasiliver-Shamis *et al*. in this volume for insight into VS formation in the target T cell. In addition, cell-to-cell spread other retroviruses will only be considered where it provides insight into the HIV-1 T cell VS.

## 2. Polarization at the HIV-1 virological synapse

**Figure 1:**
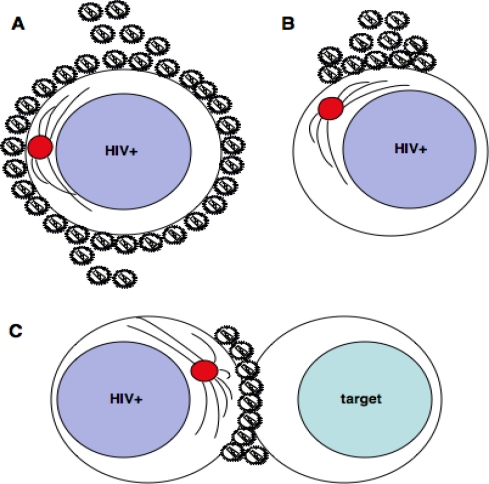
**HIV-1 egress from infected T cells.** HIV-1 assembles at and buds from the plasma membrane of infected T cells. In the absence of cell-cell contact (A and B) nascent, infectious virions could be released either by multidirectional budding from many sites of the plasma membrane (A), or by unidirectional budding from clustered viral assembly platforms (B). There is no evidence to date to suggest that the MTOC (red) is polarized at sites of HIV-1 budding in the absence of cell-cell contact. During cell-cell contact (C) the MTOC reorients in the infected T cell to the VS and there is polarization of HIV-1 Env, Gag and lipid rafts to the contact site. This directs virus budding towards the engaged target cell for efficient dissemination. Theoretically, polarized budding of HIV-1 from T cells not engaged in a VS (B) could occur in either of the following ways: if the T cell becomes activated and develops a polarized phenotype; or if HIV-1 infection drives coalescence of virus assembly domains (e.g. Gag multimerization driving lipid raft clustering at the plasma membrane); or if the infected cell is induced to polarize at the VS and subsequently separates from the other synaptic cell.

In its simplest definition, the VS is as a multimolecular structure that forms following physical contact between a retrovirus-infected cell and an uninfected target cell to facilitate virus transmission [23] (Figure 1). For HIV-1 infected T cells, this is characterized by the following features:

- Formation of a stable intercellular junction between an infected cell and an uninfected target cell [17,24–26].
- Recruitment of HIV-1 Env and the core protein (Gag) on the infected T cell, and CD4 and co-receptor (CXCR4 or CCR5) on the target T cell to sites of cell-cell contact [9,17,24,26–28].
- Co-enrichment of the integrins LFA-1, ICAM-1 and ICAM-3 [9,17,29].
- Clustering of GM1-rich lipid rafts on the infected T cell at sites of cell-cell contact [9,30].
- Remodeling of the cellular actin cytoskeleton (in both the target and the infected cell) and polarization of the microtubule organizing center (MTOC) within the infected T cell to the VS [17,28,30–32].
- Polarized virus assembly and budding of virions towards the engaged target cell [9,17,26,27].
- Rapid infection of the target cell that is estimated to be one or more orders of magnitude more efficient that equivalent cell-free infection [24–26,33].

### 2.1. Polarization of receptors at the virological synapse

T cells do not usually form stable interactions with other T cells but when homotypic contacts do occur, they are usually transient (<10 minutes)[34]. By contrast, when T cells are infected with HIV-1, T cell contacts are more long-lived and *in vitro* imaging experiments have estimated that they persist for an average of 60 minutes [17,24,26,27] with some conjugates remaining stable for several hours [24]. The driving forces behind stability of HIV-1-induced T cell-T cell contacts and VS formation is the binding of HIV-1 Env expressed on the surface of infected T cells to CD4 and coreceptor expressed on the target cell [17,24]. Env-receptor interactions are essential to trigger co-polarization of viral Env and cellular receptors – the hallmarks of VS formation – and preventing these interactions with neutralizing monoclonal antibodies or other antagonists reduces conjugate formation and blocks receptor recruitment, as evidenced by reduction of Env, Gag, CD4 and coreceptor clustering [17,24,26,35]. In addition, interactions between leukocyte function antigen-1 (LFA-1) and its cognate ligands intercellular adhesion molecule-1 and -3 (ICAM-1 and ICAM-3) provide extra stability at the VS and may also initiate outside-in signaling to trigger plasma membrane remodeling and receptor recruitment [17,29,36]. Similarly therefore, interfering with LFA-1-ICAM binding using blocking monoclonal antibodies, inhibitory peptides or T cells expressing mutated conformational forms of LFA-1 also alters conjugate stability, VS formation and HIV-1 transmission by cell-cell spread [9,29]. Polarization of viral and cellular proteins during intimate physical contact between infected and uninfected T cells serves two related purposes. Firstly, receptor binding provides the trigger for VS formation, with additional receptor recruitment further stabilizing the interface and contributing to the characteristic remodeling at the contact site. Secondly, polarization of viral proteins at cell-cell junctions provides a focus for the directed assembly and egress of HIV-1, promoting rapid and efficient infection of target cells. The presence of viral assembly platforms at the plasma membrane of infected T cells serves to integrate viral proteins, the viral genome and host cellular machinery in a coordinated manner to achieve the efficient production of nascent, infectious virus. In this way, polarization of viral and cellular proteins at the VS ensures that the necessary building blocks are delivered to the appropriate site on the infected T cell at the right time (Figure 1).

### 2.2. Polarization of HIV-1 budding

**Figure 2:**
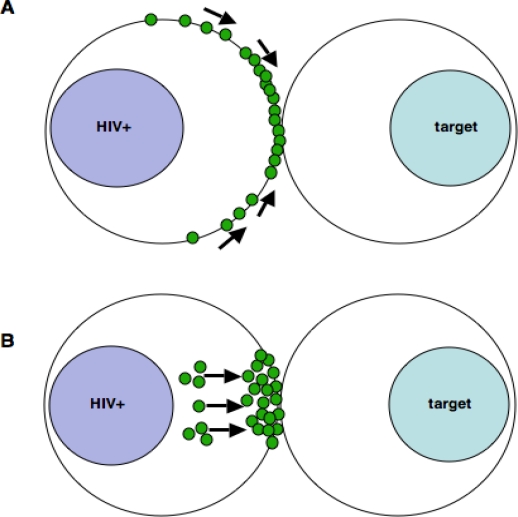
**Possible mechanisms of viral protein recruitment to the VS.** Polarization of HIV-1 assembly and budding at the VS necessitates the recruitment of viral proteins to the contact site. This could occur either by the lateral movement of plasma membrane associated viral proteins (green) due to cytoskeletal remodeling, leading to the recruitment of lipid rafts, Env and Gag to the VS and/or by Gag multimerization driving raft coalescence as suggested in Figure 1. Alternatively, activation of the regulated secretory pathway and polarization of trafficking could transport HIV-1 proteins as vesicular cargo from intracellular sites to the plasma membrane at the VS. These processes are not mutually exclusive and it is possible that at different times both pathways of HIV-1 recruitment are active.

HIV-1 assembly takes place in GM-1 rich lipid rafts at the plasma membrane of infected cells [37–44]. Concomitant with the enrichment of HIV-1 Env and Gag at the VS, lipid rafts are polarized on infected T cells to sites of cell-cell contact. Polarization of Env, Gag and lipid rafts appears to be a necessary step in cell-cell spread since disrupting lipid raft integrity by depleting membrane cholesterol with methyl-β-cyclodextran abolishes synapse formation [30]. The molecular mechanisms that trigger raft polarization are ill-defined at present and whether capping of rafts at the VS reflects the specific recruitment of membrane microdomains to the cell-cell contact site, or whether oligomerization of capsid (Gag) at sites of virus assembly at the conjugate interface drives raft coalescence, is unclear [43] (Figure 2). As coordinators of signaling platforms, lipid rafts are also recruited to other immune cell contacts such as immunological synapses (IS)[45–49] that form without the requirement for retrovirus infection of the T cell. Whether they play a more active role at the VS by recruiting down-stream signaling molecules to the contact site is an interesting proposition and further work will undoubtedly delineate the role of lipid rafts in this context.

At the plasma membrane, HIV-1 assembly is rapid, in the order of minutes [50], and HIV-1 assembly at the VS takes place with similar kinetics [27]. Recently, live cell imaging of T cell contacts has shed light on the dynamics of polarized virus assembly at the VS and has documented the appearance of synaptic accumulations or “buttons” of Gag, indicative of new particle assembly at contact sites [27]. Gag^+^ synaptic buttons appear within minutes, and are formed either by the movement of distal membrane-associated Gag towards the contact site or by coalescence of Gag protein (already at the contact site) into pre-existing buttons. The movement of Gag is rapid (averaging 0.1–0.25 μm/s) and directional, reflecting the strong probability that active transport is occurring [27]. At the VS, the frequency of contacts showing polarization of viral and cellular proteins and the relative amount of viral protein at the interface increases as a function of time [17,24,27]. Adhesion precedes polarization at the VS suggesting that viral proteins are actively mobilized towards the contact site (Figure 2). Cell-to-cell spread of HIV-1 can be severely impaired by treating cells with pharmacological inhibitors of actin remodeling or with microtubule depolymerizing agents [31] and immunofluorescence imaging has demonstrated that disrupting the integrity of the cellular cytoskeleton abolishes polarization of viral proteins at the VS by a mechanism that may involve destabilizing lipid raft-enriched virus assembly platforms [31] and/or by interfering with cytoskeletal mediated trafficking of viral proteins to sites of cell-cell contact, although this remains to be formally demonstrated. Intriguingly, the related retrovirus Murine Leukemia Virus (MLV) also shows preferential (>50-fold) *de novo* assembly at sites of cell-cell contact [51]. While the latter study was performed using epithelial cells (an inherently polarized cell type), collectively these data suggest that polarization of viral production at sites of cell-cell contact may be generalized feature of retroviral dissemination.

The molecular mechanisms that direct of polarization of HIV-1 during cell-cell transmission are currently the focus of much research. The similarities between directional HIV-1 egress at the VS and polarized secretion at the immunological synapse are tantalizing and suggest that HIV-1 may utilize pre-existing trafficking pathways that normally operate during immune cell communication to spread from one cell to another. Already, much work on the VS indicates that the enrichment of receptors, integrins and lipid rafts to T cell junctions is phenotypically reminiscent of T cell polarization at the IS, a process that is intimately linked with cytoskeletal remodeling and changes in cell shape. Below, I discuss our current understanding of the contribution of the cellular cytoskeleton in infected T cells at the VS and compare what we know to the elegant work that has been performed on the IS. We will then consider how lessons from T cell secretion at the IS may guide us towards achieving greater insight into cell-to-cell spread of HIV-1 at the VS.

### 2.3. Cytoskeletal polarization

**Figure 3:**
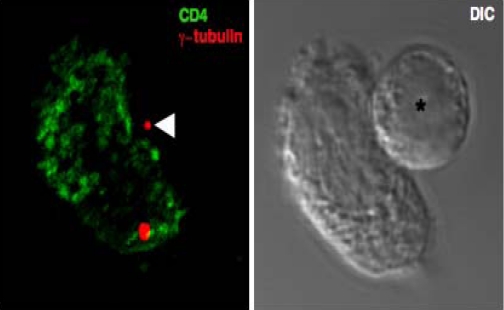
**Polarization of the microtubule organizing centre at the site of cell-cell contact.** A 3D reconstructed immunofluorescence image taken of a conjugate formed between an uninfected primary T cell pre-stained with a non-blocking CD4 monoclonal antibody (green) and an HIV-1 infected primary T cell (asterisk). The MTOC (red) was stained with a γ-tubulin specific antiserum and the polarized MTOC in the infected T cell is indicated with an arrow. The corresponding DIC image is also shown.

Polarization of HIV-1 egress at the VS almost certainly requires the active transport of viral proteins to sites of cell-cell contact mediated by the cellular cytoskeleton. A compelling feature of polarization at the VS is reorientation of the MTOC within the infected T cell to the site of cell-cell contact ([9,15,28,32,36,52,53] (Figure 3). Localization of the MTOC proximal to the VS occurs at a frequency greater than would be expected by chance [15,28,32,36] indicating that T cell contacts between retrovirus-infected and uninfected cells may be stimulating MTOC reorientation. Put simply, MTOC polarization may contribute directly to the delivery of viral proteins to the HIV-1 VS. MTOC polarization at immune cell contacts is essential for the secretion of effector proteins at the IS [54,55] and follows a series of increasingly well-defined steps. For example, one molecule in particular, ZAP-70 is a regulator of T cell activation and IS formation that operates early after T cell receptor (TCR) engagement to initiate cytoskeletal remodeling and MTOC polarization and to direct effector functions [56]. Notably, T cells defective for ZAP-70 are less able to support T cell-T cell spread of HIV-1 due to impaired VS formation and mislocalization of Gag [28], hinting that MTOC mobilization to sites of cell-cell contact may have important consequences for HIV-1 pathogenesis. Moreover, we know that treating HIV-1 infected T cells with pharmacological inhibitors of actin [26,31] and microtubule [31,33] remodeling reduces cell-to-cell spread at the VS, suggesting that maintaining a functional cytoskeleton is crucial for polarization of HIV-1 assembly at the contact site. Whether recruitment of the MTOC towards sites of cell-cell contact is itself an essential step in the sequence of viral egress at the VS, or whether a functional cytoskeleton is simply required to maintain the integrity of viral assembly platforms at the plasma membrane *per se* remains to be determined.

With this in mind, it is useful to consider the description of HIV-1 polysynapses – conjugates that form between a single infected T cell and multiple uninfected targets. During polysynapse formation, Gag accumulates in the infected T cell at interfaces formed with multiple attached target cells [9]. In this case, the MTOC can only be polarized towards a single target at any given time, or remain unpolarized. Thus, polysynapses provide us with a paradox that suggests that MTOC polarization may be dispensable at the HIV-1 T cell VS. Three possible explanations could reconcile these data. First, if Gag accumulation occurs independently of MTOC reorientation in the infected T cell, and polarization of viral proteins and the MTOC to the VS are not related and can be functionally uncoupled. Second, if MTOC polarization is still functionally linked to directional egress but precedes Gag accumulation. Third, that the first contact between an infected and an uninfected target cell leads to reorientation of the MTOC and polarization of viral budding, but that infectious virions then remain associated at the cell surface and can move around the plasma membrane to infect other target cells, similar to the ‘biofilm-like’ structures observed for HTLV [57]. Because the aforementioned studies were all performed on fixed cells, the dynamics of MTOC remodeling and Gag delivery could not be temporally and spatially evaluated. In addition, it is possible that MTOC dynamics influence the outcome of VS formation (similar to the cytotoxic IS) such that the target cell in closest proximity to the polarized MTOC at a polysynapse would be more efficiently or preferentially infected compared to neighboring cells.

Future work in this area would ideally be in the form of live-cell imaging of T cells expressing fluorescent cytoskeletal proteins to follow the kinetics of actin and microtubule remodeling following cell-cell contact. Moreover, at the CD8^+^ T cell IS MTOC docking is associated with clearance of the actin-binding protein IQGAP from the synapse [54] and it would be informative to determine if MTOC recruitment to the VS involves remodeling of IQGAP and actin-associated proteins as well. Finally, RNAi knockdown of cytoskeletal effectors such as the forming family members FMNL1 (forminlike 1), Dia1 (diaphanous 1), ADAP and IQGAP block MTOC polarization at the T cell IS [58,59] and it would be telling evaluate the effects of protein knockdown on T polarization at the VS, the recruitment of HIV-1 Env and Gag to the VS and cell-cell spread. Such experiments are likely to provide important insight into whether MTOC polarization following T cell contact is really a necessary step in directing HIV-1 protein trafficking and budding at the VS, or whether it is merely coincidental.

### 2.4. What triggers cytoskeletal polarization at the VS?

As adhesion molecules, integrins are conductors of cell-cell interactions. Integrins are a common thread linking the HIV-1 VS, the HTLV VS and the IS and are therefore good candidates for delivering directionality to viral egress at cell-cell contacts. The possibility that integrins have a more over-arching function at the VS than merely providing supporting adhesive forces is evidenced by several studies that implicate ICAM-1 signaling in MTOC polarization at the HTVL-1 synapse [36,52,53]. In that system, expression of the HTLV-1 transactivator protein Tax synergises with ICAM-1 signaling, via the Ras-MEK-ERK pathway, to provide directionality for cell-to-cell spread [53]. At the T cell IS, polarization is triggered by the strength of the TCR signal [60] with a contribution from integrin adhesion; by contrast to the natural killer cell IS where integrin signaling controls polarized vesicle trafficking [61–63]. Signaling events initiated by LFA-1-ICAM at the HIV-1 T cell VS have not been investigated and the triggers for polarization are unknown, but in light of experiments performed with other retroviruses and at the immunological synapse, it seems probable that integrins will be an important player in providing directionality at the HIV-1 synapse.

The second, more speculative, possibility is that the HIV-1 Env glycoprotein may itself trigger polarization of viral and cellular proteins at the VS. This could theoretically occur either by direct or indirect mechanisms. Engagement of Env with CD4 initiates evolution of the VS and numerous studies have attested to the importance of cytoplasmic motifs in Env driving Gag localization and directing polarized budding from infected cells [64–68]. Polarized assembly of MLV at sites of cell-cell contact is dependent on the cytoplasmic tail of Env and deleting the intracellular portion of Env abolishes polarization [51]. Whether this is due to direct or indirect effects of Env on the recruitment and sequestration of Gag at the contact interface is unknown. The gp41-cytoplasmic domain of Env is well-known to associate with the p17 matrix component of Gag thereby influencing Gag trafficking and directing sites of virus budding [67–75]. Certainly, there is no evidence to date that Env contains classical signaling motifs within its cytoplasmic domains that may activate Gag trafficking pathways, but the concept that Env may indirectly induce Gag accumulation at the HIV-1 virological is an interesting idea nonetheless.

## 3. Polarized trafficking and secretion from T cells: lesson from the immunological synapse

The plasma membrane of differentiated cells is divided into a series of microdomains each with specific composition of proteins and lipids. In the case of epithelial cells, polarity is inherent in the differentiation of membranes into basolateral and apical surfaces that are targeted by different cellular proteins; however, CD4^+^ T cells are not inherently polarized but become so following activation, migration and cell-cell contact (reviewed in [76–78]). Motile lymphocytes are functionally compartmentalized into the leading edge and the uropod, with the leading edge rich in sensory receptors such as chemokine receptors and adhesion molecules accumulating in the uropod. During cell-cell contact, T cell polarity is rearranged leading to loss of the uropod structure and reorientation of the MTOC towards the contact site at the IS. Because there are clear differences in membrane organization in response to polarization induced by migration and cell-cell contact, here we will only consider protein trafficking in T polarization during cell-cell contact and how this may be relevant to HIV-1.

One of the best-studied examples of cell-cell contact between immune cells is at the IS. The IS evolves following contact between antigen presenting cells (APC) and T cells and is a focal point for the directed secretion from T cells of immunomodulatory proteins such as cytokines [79–82], lytic proteins such as perforin and granzymes [83] and membrane-associated regulatory proteins including CTLA-4 and FasL [84–86] that is coordinated by the regulated secretory pathway. In general, the delivery of cellular proteins to the plasma membrane in T cells is controlled either by the constitutive secretory pathway that is active in all cells, or the regulated secretory pathway that is activated following TCR-engagement (or ionophore treatment) and is a unique property of professional secretory cells such as haematopoeitic cells. Numerous parallels exist between the IS and the VS [23], not least that polarized assembly and budding of HIV-1 at the T cell synapse is reminiscent of polarized exocytosis at the IS. Exocytosis from T cells at the IS has been the focus of much research and is likely to provide useful insight into the molecular mechanisms of HIV-1 protein trafficking and egress following cell-cell contact. One well characterized example of regulated secretion occurs at the cytotoxic CD8^+^ T cell IS where lytic granules (a form of specialized secretory lysosomes, SL) traffic cytolytic proteins to the plasma membrane for target cell killing [83,87]. In general, the IS is a focal point for T cell secretion with polarization of the MTOC towards the plasma membrane repositioning the Golgi apparatus, recycling compartments and associated organelles proximal to the synapse. In this way the IS serves as a “hot spot” for exocytosis and endocytosis during cell-cell contact. Lytic granules rapidly polarize towards the MTOC by moving along microtubules in a minus-end direction. Once at the MTOC, granules undergo shorter-range or tangential microtubule movement [54,88] to dock at the plasma membrane, fuse and release their contents. Studies on CTLs from human patients with genetic defects in regulated secretion and from related mouse models have identified the cellular proteins Lyst, AP-3, Rab27a and Munc13-4 as effectors of SL biogenesis, trafficking and secretion [89–95].

CD4^+^ T cells also contain SL-like compartments that help regulate the local surface expression of CTLA-4 at the IS [84,85] and also contribute to target cell killing by a small subset of CD4^+^ T cells with cytolytic function [86,96–101]. Interestingly, HIV-1 Env has been shown to colocalize with CTLA-4 in infected CD4^+^ T cells indicating that the trafficking of these proteins may intersect [102]. Regulated secretion by CD4^+^ T cells is likely to be more complex than secretion by CD8^+^ T cells and is comparatively less well-studied, not least because CD4^+^ T cells secrete a range of different effectors following stimulation by cell-cell contact. At the IS, the major function of CD4^+^ T cell secretion is the release of cytokines and the local upregulation of inhibitory proteins including CTLA-4. Cytokine secretion from CD4^+^ T involves at least two distinct pathways; a unidirectional pathway that focuses secretion of cytokines such as IL-2 and interferon-γ towards the engaged target cell, and a multidirectional pathway for the release of proinflammatory proteins like TNF [82]. In an effort to identify trafficking proteins implicit in CD4^+^ cell secretion, Huse *et al.* generated a panel of GFP-fusion protein and assessed their localization with cytokines and identified Rab3d and Rab19 (involved in synaptic cytokine secretion), syntaxin 6 (multidirectional secretion) and Rab37 and Vti1b (intermediate effect) as candidates for CD4^+^ T cell secretion. Crucially, these Rabs and SNARES are not the same effectors implicated in CD8^+^ T cell secretion. Furthermore, secretion from CD4^+^ T cells has different requirements for microtubule-based movement and actin-effector protein function depending on the nature of the secreted cargo and the directionality of secretion [82,103]. Whether HIV-1 protein trafficking to the VS intersects pre-existing pathways that target proteins to the IS in T cells is not known, but it seems probable that there will be overlap between contact-induced assembly of HIV-1 proteins at the VS and regulated secretion from T cells.

In addition to exocytosis, the IS as an active site of endocytosis that helps regulates the local expression of the TCR and to recycle endocytosed proteins back to sites of cell-cell contact [63,104]. Endocytosis of HIV-1 proteins at the VS is a potential mechanism by which viral proteins could be targeted to secretory compartments (SL and/or recycling endosomes) for delivery back to the plasma membrane at the contact site. One might imagine that this could occur either by sorting of viral proteins from early endosomes into SL or alternatively by delivery to recycling endosomes that polarize at T cell synapses. For example, HIV-1 Env contains both dileucine and tyrosine motifs that are recognized by clathrin adapators AP-1 and AP-2 [105,106]. Moreover, potential endocytosis at the VS might also provide a necessary stop signal to trigger the dissociation of cell-cell contacts [24] by regulating the local levels of surface receptors, but this is purely speculative.

## 4. Concluding remarks

Polarization of HIV-1 assembly and budding at the T cell VS is central to promoting efficient HIV-1 dissemination and enhancing viral pathogenesis, as many studies attest. T cells are well placed to respond to external stimuli and can rapidly adopt a polarized membrane morphology and align the cellular cytoskeleton in response to cell-cell contact, most notably at the immunological synapse. Cell-cell spread of HIV-1 at the VS is a relatively new and striking example of T cell polarization that takes place at immune cell contacts in the context of retroviral infection. Future work in this area will undoubtedly attempt to unravel the pathway of protein trafficking to the VS. Specifically, it is timely to explore the molecular regulation of polarization at the VS and to determine to what extent protein trafficking to the VS overlaps with the better defined pathway of T cell secretion at the IS. A panel of effector proteins required for polarized secretion at the IS have now been described in CD8^+^ and CD4^+^ T cells and this seems a good starting point. Such studies will not only provide further insight into the cell biology of retroviral infection, but may also identifying novel drug targets to inhibit cell-cell spread of HIV-1.

## References

[1] Sattentau Q (2008). Avoiding the void: cell-to-cell spread of human viruses. Nat Rev Microbiol.

[2] Pearce-Pratt R, Malamud D, Phillips DM (1994). Role of the cytoskeleton in cell-to-cell transmission of human immunodeficiency virus. J Virol.

[3] Phillips DM, Bourinbaiar AS (1992). Mechanism of HIV spread from lymphocytes to epithelia. Virology.

[4] Phillips DM, Tan X, Perotti ME, Zacharopoulos VR (1998). Mechanism of monocyte-macrophage-mediated transmission of HIV. AIDS Res Hum Retroviruses.

[5] Perotti ME, Tan X, Phillips DM (1996). Directional budding of human immunodeficiency virus from monocytes. J Virol.

[6] Fais S, Capobianchi M, Abbate I, Castilletti C, Gentile M, Fei P, Ameglio F, Dianzani F (1995). Unidirectional budding of HIV-1 at the site of cell-to-cell contact is associated with co-polarization of intercellular adhesion molecules and HIV-1 viral matrix protein. AIDS.

[7] Sattentau QJ, Moore JP (1993). The role of CD4 in HIV binding and entry. Philos Trans R Soc Lond B Biol Sci.

[8] Schacker T, Little S, Connick E, Gebhard K, Zhang ZQ, Krieger J, Pryor J, Havlir D, Wong JK, Schooley RT, Richman D, Corey L, Haase AT (2001). Productive infection of T cells in lymphoid tissues during primary and early human immunodeficiency virus infection. J Infect Dis.

[9] Rudnicka D, Feldmann J, Porrot F, Wietgrefe S, Guadagnini S, Prevost MC, Estaquier J, Haase AT, Sol-Foulon N, Schwartz O (2009). Simultaneous cell-to-cell transmission of human immunodeficiency virus to multiple targets through polysynapses. J Virol.

[10] Brenchley JM, Schacker TW, Ruff LE, Price DA, Taylor JH, Beilman GJ, Nguyen PL, Khoruts A, Larson M, Haase AT, Douek DC (2004). CD4+ T cell depletion during all stages of HIV disease occurs predominantly in the gastrointestinal tract. J Exp Med.

[11] Guadalupe M, Reay E, Sankaran S, Prindiville T, Flamm J, McNeil A, Dandekar S (2003). Severe CD4+ T-cell depletion in gut lymphoid tissue during primary human immunodeficiency virus type 1 infection and substantial delay in restoration following highly active antiretroviral therapy. J Virol.

[12] Mehandru S, Poles MA, Tenner-Racz K, Horowitz A, Hurley A, Hogan C, Boden D, Racz P, Markowitz M (2004). Primary HIV-1 infection is associated with preferential depletion of CD4+ T lymphocytes from effector sites in the gastrointestinal tract. J Exp Med.

[13] Veazey RS, DeMaria M, Chalifoux LV, Shvetz DE, Pauley DR, Knight HL, Rosenzweig M, Johnson RP, Desrosiers RC, Lackner AA (1998). Gastrointestinal tract as a major site of CD4+ T cell depletion and viral replication in SIV infection. Science.

[14] Schacker T, Little S, Connick E, Gebhard-Mitchell K, Zhang Z-Q, Krieger J, Pryor J, Havlir D, Wong J, Richman D, Corey L, Haase A (2000). Rapid accumulation of human immunodeficiency virus (HIV) in lymphatic tissue reservoirs during acute and early HIV infection: implications for timing of antiretroviral therapy. J Inf Dis.

[15] Igakura T, Stinchcombe JC, Goon PK, Taylor GP, Weber JN, Griffiths GM, Tanaka Y, Osame M, Bangham CR (2003). Spread of HTLV-I between lymphocytes by virus-induced polarization of the cytoskeleton. Science.

[16] Jolly CL, Sattentau QJ (2002). HIV Env induces the formation of supramolecular activation structures in CD4+ T cells. Mol Biol Cell.

[17] Jolly C, Kashefi K, Hollinshead M, Sattentau QJ (2004). HIV-1 cell to cell transfer across an Env-induced, actin-dependent synapse. J Exp Med.

[18] McDonald D, Wu L, Bohks SM, KewalRamani VN, Unutmaz D, Hope TJ (2003). Recruitment of HIV and its receptors to dendritic cell-T cell junctions. Science.

[19] Groot F, Welsch S, Sattentau QJ (2008). Efficient HIV-1 transmission from macrophages to T cells across transient virological synapses. Blood.

[20] Gousset K, Ablan SD, Coren LV, Ono A, Soheilian F, Nagashima K, Ott DE, Freed EO (2008). Real-time visualization of HIV-1 GAG trafficking in infected macrophages. PLoS Pathog.

[21] Alfsen A, Yu H, Magerus-Chatinet A, Schmitt A, Bomsel M (2005). HIV-1-infected blood mononuclear cells form an integrin- and agrin-dependent viral synapse to induce efficient HIV-1 transcytosis across epithelial cell monolayer. Mol Biol Cell.

[22] Moore JP, Kitchen SG, Pugach P, Zack JA (2004). The CCR5 and CXCR4 coreceptors--central to understanding the transmission and pathogenesis of human immunodeficiency virus type 1 infection. AIDS Res Hum Retroviruses.

[23] Jolly C, Sattentau QJ (2004). Retroviral spread by induction of virological synapses. Traffic.

[24] Martin N, Welsch S, Jolly C, Briggs JA, Vaux D, Sattentau QJ (2010). Virological Synapse-Mediated Spread of Human Immunodeficiency Virus Type-1 between T cells is Sensitive to Entry Inhibition. J Virol.

[25] Sourisseau M, Sol-Foulon N, Porrot F, Blanchet F, Schwartz O (2007). Inefficient human immunodeficiency virus replication in mobile lymphocytes. J Virol.

[26] Chen P, Hubner W, Spinelli MA, Chen BK (2007). Predominant mode of human immunodeficiency virus transfer between T cells is mediated by sustained Env-dependent neutralization-resistant virological synapses. J Virol.

[27] Hubner W, McNerney GP, Chen P, Dale BM, Gordon RE, Chuang FY, Li XD, Asmuth DM, Huser T, Chen BK (2009). Quantitative 3D video microscopy of HIV transfer across T cell virological synapses. Science.

[28] Sol-Foulon N, Sourisseau M, Porrot F, Thoulouze MI, Trouillet C, Nobile C, Blanchet F, di Bartolo V, Noraz N, Taylor N, Alcover A, Hivroz C, Schwartz O (2007). ZAP-70 kinase regulates HIV cell-to-cell spread and virological synapse formation. Embo J.

[29] Jolly C, Mitar I, Sattentau QJ (2007). Adhesion molecule interactions facilitate human immunodeficiency virus type 1-induced virological synapse formation between T cells. J Virol.

[30] Jolly C, Sattentau QJ (2005). Human immunodeficiency virus type 1 virological synapse formation in T cells requires lipid raft integrity. J Virol.

[31] Jolly C, Mitar I, Sattentau QJ (2007). Requirement for an intact T cell actin and tubulin cytoskeleton for efficient HIV-1 assembly and spread. J Virol.

[32] Jolly C, Sattentau Q (2010). University College London and The University of Oxford.

[33] Mazurov D, Ilinskaya A, Heidecker G, Lloyd P, Derse D (2010). Quantitative comparison of HTLV-1 and HIV-1 cell-to-cell infection with new replication dependent vectors. PLoS Pathog.

[34] Sabatos CA, Doh J, Chakravarti S, Friedman RS, Pandurangi PG, Tooley AJ, Krummel MF (2008). A synaptic basis for paracrine interleukin-2 signaling during homotypic T cell interaction. Immunity.

[35] Puigdomenech I, Massanella M, Izquierdo-Useros N, Ruiz-Hernandez R, Curriu M, Bofill M, Martinez-Picado J, Juan M, Clotet B, Blanco J (2008). HIV transfer between CD4 T cells does not require LFA-1 binding to ICAM-1 and is governed by the interaction of HIV envelope glycoprotein with CD4. Retrovirology.

[36] Barnard AL, Igakura T, Tanaka Y, Taylor GP, Bangham CR (2005). Engagement of specific T-cell surface molecules regulates cytoskeletal polarization in HTLV-1-infected lymphocytes. Blood.

[37] Bhattacharya J, Peters PJ, Clapham PR (2004). Human immunodeficiency virus type 1 envelope glycoproteins that lack cytoplasmic domain cysteines: impact on association with membrane lipid rafts and incorporation onto budding virus particles. J Virol.

[38] Ding L, Derdowski A, Wang JJ, Spearman P (2003). Independent segregation of human immunodeficiency virus type 1 Gag protein complexes and lipid rafts. J Virol.

[39] Campbell SM, Crowe SM, Mak J (2001). Lipid rafts and HIV-1: from viral entry to assembly of progeny virions. J Clin Virol.

[40] Rousso I, Mixon MB, Chen BK, Kim PS (2000). Palmitoylation of the HIV-1 envelope glycoprotein is critical for viral infectivity. Proc Natl Acad Sci U S A.

[41] Ono A, Freed EO (2001). Plasma membrane rafts play a critical role in HIV-1 assembly and release. Proc Natl Acad Sci U S A.

[42] Nguyen DH, Hildreth JE (2000). Evidence for budding of human immunodeficiency virus type 1 selectively from glycolipid-enriched membrane lipid rafts. J Virol.

[43] Lindwasser OW, Resh MD (2001). Multimerization of human immunodeficiency virus type 1 Gag promotes its localization to barges, raft-like membrane microdomains. J Virol.

[44] Holm K, Weclewicz K, Hewson R, Suomalainen M (2003). Human immunodeficiency virus type 1 assembly and lipid rafts: Pr55(gag) associates with membrane domains that are largely resistant to Brij98 but sensitive to Triton X-100. J Virol.

[45] Xavier R, Brennan T, Li Q, McCormack C, Seed B (1998). Membrane compartmentation is required for efficient T cell activation. Immunity.

[46] Ilangumaran S, He HT, Hoessli DC (2000). Microdomains in lymphocyte signalling: beyond GPI-anchored proteins. Immunol Today.

[47] Montixi C, Langlet C, Bernard AM, Thimonier J, Dubois C, Wurbel MA, Chauvin JP, Pierres M, He HT (1998). Engagement of T cell receptor triggers its recruitment to low-density detergent-insoluble membrane domains. Embo J.

[48] Harder T (2004). Lipid raft domains and protein networks in T-cell receptor signal transduction. Curr Opinion Immunol.

[49] Manes S, Viola A (2006). Lipid rafts in lymphocyte activation and migration. Mol Membr Biol.

[50] Jouvenet N, Bieniasz PD, Simon SM (2008). Imaging the biogenesis of individual HIV-1 virions in live cells. Nature.

[51] Jin J, Sherer NM, Heidecker G, Derse D, Mothes W (2009). Assembly of the murine leukemia virus is directed towards sites of cell-cell contact. PLoS Biol.

[52] Nejmeddine M, Barnard AL, Tanaka Y, Taylor GP, Bangham CR (2005). Human T-lymphotropic Virus, Type 1, Tax Protein Triggers Microtubule Reorientation in the Virological Synapse. J Biol Chem.

[53] Nejmeddine M, Negi VS, Mukherjee S, Tanaka Y, Orth K, Taylor GP, Bangham CR (2009). HTLV-1-Tax and ICAM-1 act on T-cell signal pathways to polarize the microtubule-organizing center at the virological synapse. Blood.

[54] Stinchcombe JC, Majorovits E, Bossi G, Fuller S, Griffiths GM (2006). Centrosome polarization delivers secretory granules to the immunological synapse. Nature.

[55] Kupfer A, Dennert G, Singer SJ (1983). Polarization of the Golgi apparatus and the microtubule-organizing center within cloned natural killer cells bound to their targets. Proc Natl Acad Sci U S A.

[56] Blanchard N, Di Bartolo V, Hivroz C (2002). In the immune synapse, ZAP–70 controls T cell polarization and recruitment of signaling proteins but not formation of the synaptic pattern. Immunity.

[57] Pais-Correia AM, Sachse M, Guadagnini S, Robbiati V, Lasserre R, Gessain A, Gout O, Alcover A, Thoulouze MI (2010). Biofilm-like extracellular viral assemblies mediate HTLV-1 cell-to-cell transmission at virological synapses. Nat Med.

[58] Combs J, Kim SJ, Tan S, Ligon LA, Holzbaur EL, Kuhn J, Poenie M (2006). Recruitment of dynein to the Jurkat immunological synapse. Proc Natl Acad Sci U S A.

[59] Gomez TS, Kumar K, Medeiros RB, Shimizu Y, Leibson PJ, Billadeau DD (2007). Formins regulate the actin-related protein 2/3 complex-independent polarization of the centrosome to the immunological synapse. Immunity.

[60] Jenkins MR, Tsun A, Stinchcombe JC, Griffiths GM (2009). The strength of T cell receptor signal controls the polarization of cytotoxic machinery to the immunological synapse. Immunity.

[61] Barber DF, Faure M, Long EO (2004). LFA-1 contributes an early signal for NK cell cytotoxicity. J Immunol.

[62] Bryceson YT, March ME, Barber DF, Ljunggren HG, Long EO (2005). Cytolytic granule polarization and degranulation controlled by different receptors in resting NK cells. J Exp Med.

[63] Liu D, Bryceson YT, Meckel T, Vasiliver-Shamis G, Dustin ML, Long EO (2009). Integrin-dependent organization and bidirectional vesicular traffic at cytotoxic immune synapses. Immunity.

[64] Deschambeault J, Lalonde JP, Cervantes-Acosta G, Lodge R, Cohen EA, Lemay G (1999). Polarized human immunodeficiency virus budding in lymphocytes involves a tyrosine-based signal and favors cell-to-cell viral transmission. J Virol.

[65] Owens RJ, Dubay JW, Hunter E, Compans RW (1991). Human immunodeficiency virus envelope protein determines the site of virus release in polarized epithelial cells. Proc Natl Acad Sci U S A.

[66] Day JR, Munk C, Guatelli JC (2004). The membrane-proximal tyrosine-based sorting signal of human immunodeficiency virus type 1 gp41 is required for optimal viral infectivity. J Virol.

[67] Lodge R, Lalonde JP, Lemay G, Cohen EA (1997). The membrane-proximal intracytoplasmic tyrosine residue of HIV-1 envelope glycoprotein is critical for basolateral targeting of viral budding in MDCK cells. Embo J.

[68] Lodge R, Gottlinger H, Gabuzda D, Cohen EA, Lemay G (1994). The intracytoplasmic domain of gp41 mediates polarized budding of human immunodeficiency virus type 1 in MDCK cells. J Virol.

[69] Hourioux C, Brand D, Sizaret PY, Lemiale F, Lebigot S, Barin F, Roingeard P (2000). Identification of the glycoprotein 41(TM) cytoplasmic tail domains of human immunodeficiency virus type 1 that interact with Pr55Gag particles. AIDS Res Hum Retroviruses.

[70] Cosson P (1996). Direct interaction between the envelope and matrix proteins of HIV-1. Embo J.

[71] Ono A, Huang M, Freed EO (1997). Characterization of human immunodeficiency virus type 1 matrix revertants: effects on virus assembly, Gag processing, and Env incorporation into virions. J Virol.

[72] Freed EO, Martin MA (1996). Domains of the human immunodeficiency virus type 1 matrix and gp41 cytoplasmic tail required for envelope incorporation into virions. J Virol.

[73] Freed EO, Martin MA (1995). Virion incorporation of envelope glycoproteins with long but not short cytoplasmic tails is blocked by specific, single amino acid substitutions in the human immunodeficiency virus type 1 matrix. J Virol.

[74] Yu X, Yuan X, Matsuda Z, Lee TH, Essex M (1992). The matrix protein of human immunodeficiency virus type 1 is required for incorporation of viral envelope protein into mature virions. J Virol.

[75] Murakami T, Freed EO (2000). Genetic evidence for an interaction between human immunodeficiency virus type 1 matrix and alpha-helix 2 of the gp41 cytoplasmic tail. J Virol.

[76] Sanchez-Madrid F, del Pozo M (1999). Leukocyte polarization in cell migration and immune interactions. EMBO J.

[77] Billadeau DD, Nolz JC, Gomez TS (2007). Regulation of T-cell activation by the cytoskeleton. Nat Rev Immunol.

[78] Krummel MF, Macara I (2006). Maintenance and modulation of T cell polarity. Nat Immunol.

[79] Poo WJ, Conrad L, Janeway CA (1988). Receptor-directed focusing of lymphokine release by helper T cells. Nature.

[80] Kupfer H, Monks CR, Kupfer A (1994). Small splenic B cells that bind to antigen-specific T helper (Th) cells and face the site of cytokine production in the Th cells selectively proliferate: immunofluorescence microscopic studies of Th-B antigen-presenting cell interactions. J Exp Med.

[81] Kupfer A, Mosmann TR, Kupfer H (1991). Polarized expression of cytokines in cell conjugates of helper T cells and splenic B cells. Proc Natl Acad Sci U S A.

[82] Huse M, Lillemeier BF, Kuhns MS, Chen DS, Davis MM (2006). T cells use two directionally distinct pathways for cytokine secretion. Nat Immunol.

[83] Peters PJ, Borst J, Oorschot V, Fukuda M, Krahenbuhl O, Tschopp J, Slot JW, Geuze HJ (1991). Cytotoxic T lymphocyte granules are secretory lysosomes, containing both perforin and granzymes. J Exp Med.

[84] Linsley PS, Bradshaw J, Greene J, Peach R, Bennett KL, Mittler RS (1996). Intracellular trafficking of CTLA-4 and focal localization towards sites of TCR engagement. Immunity.

[85] Iida T, Ohno H, Nakaseko C, Sakuma M, Takeda-Ezaki M, Arase H, Kominami E, Fujisawa T, Saito T (2000). Regulation of cell surface expression of CTLA-4 by secretion of CTLA-4-containing lysosomes upon activation of CD4+ T cells. J Immunol.

[86] Bossi G, Griffiths GM (1999). Degranulation plays an essential part in regulating cell surface expression of Fas ligand in T cells and natural killer cells. Nat Med.

[87] Stinchcombe JC, Bossi G, Booth S, Griffiths GM (2001). The immunological synapse of CTL contains a secretory domain and membrane bridges. Immunity.

[88] Beal AM, Anikeeva N, Varma R, Cameron TO, Vasiliver-Shamis G, Norris PJ, Dustin ML, Sykulev Y (2009). Kinetics of early T cell receptor signaling regulate the pathway of lytic granule delivery to the secretory domain. Immunity.

[89] Feldmann J, Callebaut I, Raposo G, Certain S, Bacq D, Dumont C, Lambert N, Ouachee-Chardin M, Chedeville G, Tamary H, Minard-Colin V, Vilmer E, Blanche S, Le Deist F, Fischer A, de Saint Basile G (2003). Munc13-4 is essential for cytolytic granules fusion and is mutated in a form of familial hemophagocytic lymphohistiocytosis (FHL3). Cell.

[90] Neeft M, Wieffer M, de Jong AS, Negroiu G, Metz CH, van Loon A, Griffith J, Krijgsveld J, Wulffraat N, Koch H, Heck AJ, Brose N, Kleijmeer M, van der Sluijs P (2005). Munc13-4 is an effector of rab27a and controls secretion of lysosomes in hematopoietic cells. Mol Biol Cell.

[91] Haddad EK, Wu X, Hammer JA, Henkart PA (2001). Defective granule exocytosis in Rab27a-deficient lymphocytes from Ashen mice. J Cell Biol.

[92] Stinchcombe JC, Barral DC, Mules EH, Booth S, Hume AN, Machesky LM, Seabra MC, Griffiths GM (2001). Rab27a is required for regulated secretion in cytotoxic T lymphocytes. J Cell Biol.

[93] Ward DM, Griffiths GM, Stinchcombe JC, Kaplan J (2000). Analysis of the lysosomal storage disease Chediak-Higashi syndrome. Traffic.

[94] Stinchcombe JC, Page LJ, Griffiths GM (2000). Secretory lysosome biogenesis in cytotoxic T lymphocytes from normal and Chediak Higashi syndrome patients. Traffic.

[95] Clark RH, Stinchcombe JC, Day A, Blott E, Booth S, Bossi G, Hamblin T, Davies EG, Griffiths GM (2003). Adaptor protein 3-dependent microtubule-mediated movement of lytic granules to the immunological synapse. Nat Immunol.

[96] Sun Q, Burton RL, Lucas KG (2002). Cytokine production and cytolytic mechanism of CD4(+) cytotoxic T lymphocytes in ex vivo expanded therapeutic Epstein-Barr virus-specific T-cell cultures. Blood.

[97] Susskind B, Shornick MD, Iannotti MR, Duffy B, Mehrotra PT, Siegel JP, Mohanakumar T (1996). Cytolytic effector mechanisms of human CD4+ cytotoxic T lymphocytes. Hum Immunol.

[98] Yasukawa M, Ohminami H, Arai J, Kasahara Y, Ishida Y, Fujita S (2000). Granule exocytosis, and not the fas/fas ligand system, is the main pathway of cytotoxicity mediated by alloantigen-specific CD4(+) as well as CD8(+) cytotoxic T lymphocytes in humans. Blood.

[99] Williams NS, Engelhard VH (1996). Identification of a population of CD4+ CTL that utilizes a perforin- rather than a Fas ligand-dependent cytotoxic mechanism. J Immunol.

[100] Stalder T, Hahn S, Erb P (1994). Fas antigen is the major target molecule for CD4+ T cell-mediated cytotoxicity. J Immunol.

[101] Appay V, Zaunders JJ, Papagno L, Sutton J, Jaramillo A, Waters A, Easterbrook P, Grey P, Smith D, McMichael AJ, Cooper DA, Rowland-Jones SL, Kelleher AD (2002). Characterization of CD4(+) CTLs ex vivo. J Immunol.

[102] Miranda LR, Schaefer BC, Kupfer A, Hu Z, Franzusoff A (2002). Cell surface expression of the HIV-1 envelope glycoproteins is directed from intracellular CTLA-4-containing regulated secretory granules. Proc Natl Acad Sci U S A.

[103] Morales-Tirado V, Johannson S, Hanson E, Howell A, Zhang J, Siminovitch KA, Fowell DJ (2004). Cutting edge: selective requirement for the Wiskott-Aldrich syndrome protein in cytokine, but not chemokine, secretion by CD4+ T cells. J Immunol.

[104] Das V, Nal B, Dujeancourt A, Thoulouze MI, Galli T, Roux P, Dautry-Varsat A, Alcover A (2004). Activation-induced polarized recycling targets T cell antigen receptors to the immunological synapse; involvement of SNARE complexes. Immunity.

[105] Boge M, Wyss S, Bonifacino JS, Thali M (1998). A membrane-proximal tyrosine-based signal mediates internalization of the HIV-1 envelope glycoprotein via interaction with the AP-2 clathrin adaptor. J Biol Chem.

[106] Wyss S, Berlioz-Torrent C, Boge M, Blot G, Honing S, Benarous R, Thali M (2001). The highly conserved C-terminal dileucine motif in the cytosolic domain of the human immunodeficiency virus type 1 envelope glycoprotein is critical for its association with the AP-1 clathrin adaptor [correction of adapter]. J Virol.

